# Feasibility of a Wiki as a Participatory Tool for Patients in Clinical Guideline Development

**DOI:** 10.2196/jmir.2080

**Published:** 2012-10-26

**Authors:** Elvira ME den Breejen, Willianne LDM Nelen, Jose M.L Knijnenburg, Jako S Burgers, Rosella PMG Hermens, Jan AM Kremer

**Affiliations:** ^1^Radboud University Nijmegen Medical CenterDepartment of Obstetrics and GynecologyRadboud University NijmegenNijmegenNetherlands; ^2^Dutch patient association for people with fertility problems FreyaWijchenNetherlands; ^3^Dutch college of General PractitionersUtrechtNetherlands; ^4^Radboud University Nijmegen Medical CenterDepartment of IQ HealthcareRadboud University NijmegenNijmegenNetherlands

**Keywords:** Wiki, patient participation, infertility, Web 2.0, guideline development

## Abstract

**Background:**

Patient participation is essential in developing high-quality guidelines but faces practical challenges. Evidence on timing, methods, evaluations, and outcomes of methodologies for patient participation in guideline development is lacking.

**Objective:**

To assess the feasibility of a wiki as a participatory tool for patients in the development of a guideline on infertility determined by (1) use of the wiki (number of page views and visitors), (2) benefits of the wiki (ie, number, content, and eligibility of the recommendations to be integrated into the guideline), and (3) patients’ facilitators of and barriers to adoption, and the potential challenges to be overcome in improving this wiki.

**Methods:**

To obtain initial content for the wiki, we conducted in-depth interviews (n = 12) with infertile patients. Transcripts from the interviews were translated into 90 draft recommendations. These were presented on a wiki. Over 7 months, infertile patients were invited through advertisements or mailings to formulate new or modify existing recommendations. After modifying the recommendations, we asked patients to select their top 5 or top 3 recommendations for each of 5 sections on fertility care. Finally, the guideline development group assessed the eligibility of the final set of recommendations within the scope of the guideline. We used a multimethod evaluation strategy to assess the feasibility of the wiki as a participatory tool for patients in guideline development.

**Results:**

The wiki attracted 298 unique visitors, yielding 289 recommendations. We assessed the 21 recommendations ranked as the top 5 or top 3 for their eligibility for being integrated into the clinical practice guideline. The evaluation identified some challenges needed to be met to improve the wiki tool, concerning its ease of use, website content and layout, and characteristics of the wiki tool.

**Conclusions:**

The wiki is a promising and feasible participatory tool for patients in guideline development. A modified version of this tool including new modalities (eg, automatically limiting the number and length of recommendations, using a fixed format for recommendations, including a motivation page, and adding a continuous prioritization system) should be developed and evaluated in a patient-centered design.

## Introduction

Having patients participate in clinical practice guideline (CPG) development is essential but challenging [1,2]. Their participation is particularly assumed to result in higher-quality guidelines in terms of applicability, acceptability, usefulness, and enhancement of implementation [1-7]. For instance, patient participation is one of the key criteria of the Appraisal of Guidelines Research and Evaluation (AGREE) instrument [8], which is used to assess the methodological quality of guidelines. However, only 25%-50% of CPG developers regularly involve patients [9].

Several practical limitations could explain why patient participation is not common practice in CPG development. First, various methods for patient participation in CPG development can be used, such as conducting in-depth interviews or focus group meetings to explore patients’ preferences, asking patients’ representatives to comment on drafts of the CPG, or including patients’ representatives or patients in the CPG development group [3,6,10-14]. However, practical guidance on how and when to apply these methods is lacking [15]. Second, all methods are restricted to including a selected number of patients or patients’ representatives and do not involve a large population of patients. Third, transparently integrating patients’ preferences into CPG recommendations is difficult and often unclear [16]. Fourth, organizational (eg, recruitment of participants), financial (eg, costs of patients’ education or for conducting focus groups), and sociopolitical barriers (eg, CPG developers’ resistance to including patients in the CPG group) may impede patient participation in CPG development [13]. Finally, studies on the effectiveness and impact of patient participation are limited [15]. A new methodology for patient participation in CPG development that enables overcoming most of these drawbacks is thus necessary.

Web 2.0 tools offer opportunities to let nonorganized groups participate in a complex process such as CPG development [17-20]. In particular a wiki, such as Wikipedia, seems to be an easily accessed tool, which enables patients to collaborate in formulating guideline recommendations directly. Ideally, to test the feasibility of such a new method for patient participation in CPG development, an Internet-using young target group such as infertile patients [21-23] is preferred. Infertility is commonly defined as “any form of reduced fertility with prolonged time of unwanted non-conception” [24] and affects approximately 80 million couples worldwide [25,26]. In this study, we applied wiki technology as a participatory tool for patients in the development of a multidisciplinary CPG on infertility and aimed to assess its feasibility.

## Methods

### Setting

#### Fertility Care

In the Netherlands, fertility care is mostly publically arranged and provided by various professionals. First, fertility care is provided by the general practitioner and may be part of an initial fertility assessment after a prolonged time of unwanted nonconception. Second, the general practitioner can refer couples to a gynecologist in a general (secondary care) or a university (tertiary care) hospital to complete the fertility assessment, determine a cause of infertility, and define a suitable treatment policy. Third, if a severe male factor is diagnosed, a urologist may be consulted. Furthermore, since infertility has a high emotional and psychological impact, which also interferes with work, a psychologist and occupational physician are regularly engaged in the care pathway. In vitro fertilization and intracytoplasmic sperm injection are provided by 13 licensed hospitals (8 university hospitals, 4 general hospitals, and 1 private clinic). Ovulation induction and intrauterine insemination are performed in all Dutch hospitals. Ovulation induction, intrauterine insemination cycles, and the first three in vitro fertilization or intracytoplasmic sperm injection treatment cycles are reimbursed as part of the basic health care package according to the Health Insurance Act.

#### Guideline Development

In February 2008, a collaboration of stakeholders (a general practitioner, 2 gynecologists, a urologist/sexologist, a clinical embryologist, a clinical chemical specialist, a medical psychologist, an occupational physician, 2 patients’ representatives, and a researcher) was set up to develop a national multidisciplinary paper-based CPG on infertility. CPGs are defined as sets of evidence- or consensus-based recommendations describing optimal patient care to assist health care professionals and patients in clinical decision making [2]. The aim of the CPG was to focus on organizational and patient-centered aspects of fertility care. Two representatives of the Dutch patients’ association for infertility, Freya, participated in the CPG development group. However, for direct patient participation in this guideline, we applied a wiki concurrently with this guideline development phase.

### Study Objectives

The objective of this study was to assess the feasibility of the wiki as a participatory tool for patient participation in CPG development. The feasibility of the wiki was determined by three end points: (1) use of the wiki and users’ characteristics (number of page views and visitors), (2) wiki content quality, particularly the assessment of various aspects of the final set of unique recommendations (ie, number, content, and their eligibility for integration into the CPG) for high-quality fertility care, and (3) wiki system quality (ie, patients’ facilitators of and barriers to adoption of this wiki as a participatory tool for direct patient involvement in CPG development, as well as potential suggestions for improvement).

### Wiki Tool Development

We developed a conventional wiki website using MediaWiki software and made accessible through the Freya website, called FreyaWIKI [27]. During the preparation phase, we first conducted in-depth interviews to obtain initial content for the wiki. Next, we structured the wiki tool according to the topics of the recommendations derived from the interviews.

#### Obtaining Initial Content of the Wiki Tool From In-Depth Interviews

To obtain the initial content for this wiki, we first conducted 12 semistructured in-depth interviews with infertile couples during different phases of care, from the first visit to the general practitioner, to (non)pregnant status after completing medically assisted reproduction techniques [28]. Patients visiting outpatient clinics in Nijmegen and Amsterdam were consecutively invited to participate through an information letter. Subsequently, 1 researcher (EB) obtained the final consent by telephone. Participants were asked to specify perceived bottlenecks in their fertility care pathway, using an interview guide including the main treatment stages of their fertility care pathway (eg, treatment by a general practitioner, gynecologist, or urologist). Interviews were audiotaped and transcribed verbatim. Next, a researcher (EB) and the chief executive of Freya (JK) independently translated these bottlenecks into draft patient recommendations. These draft recommendations were formulated as “I want my doctor to....” Consensus on the formulation of patient draft recommendations was reached through discussion.

#### Structuring the Wiki Tool

Division of the draft recommendations into sections and subsections determined the structure of the wiki. Draft recommendations were divided into 4 sections (EB,JK), consisting of 3 sections referring to the care delivered by the 3 most involved professionals and a general section for recommendations important to all professionals: general care, care delivered by a general practitioner, gynecological care, and urological care. To provide more structure in the wiki, the draft recommendations in each of these 4 sections were subdivided into 8 subsections (EB, JK) based on aspects of care that are known to be important to infertile patients: 3 medical-technical aspects (ie, examination, therapy, and referral), 4 patient-centered aspects (ie, organization of care, information provision, communication, and staff attitudes), and 1 general aspect (ie, general) [29]. These subsections were presented on the wiki in the following order: general (recommendations in general and those that don’t apply to other care aspects), information provision (recommendations on oral and paper-based information provision), organization (recommendations on the organization of fertility care, for example, adjustment of care between different health care professionals, accessibility of care), staff attitudes (recommendations on the attitude of health care professionals toward the patient, for example, having empathy), communication (recommendations on communication between the health care professional and the patient), examination (recommendations on examinations during fertility care), therapy (recommendations on therapy, namely infertility treatment by, for example, in vitro fertilization), and referral (recommendations on referral from one health care professional to another, for example, from a general practitioner to a gynecologist). Discrepancies in division and subdivision of the draft recommendations were resolved through discussion.

### Patient Participation in CPG Development

#### Recruiting Participants

We recruited participants for the wiki evaluation through mailings to members of Freya, the Dutch patients’ association for infertility; advertisements in Freya’s quarterly journal; links on websites of Freya and the professional societies (eg, general practitioners, gynecologists, urologists, and clinical embryologists); and links in social media (eg, Hyves, Twitter, and Facebook). In addition, we sent advertising posters to all 103 clinics offering fertility treatments in the Netherlands for their waiting rooms.

#### Obtaining Recommendations From Wiki Participants

##### Formulating Recommendations

From May to December 2008, we presented the draft recommendations for fertility care on the wiki. Patients were invited to modify or refine these recommendations and to add new recommendations. During this process, we asked patients to subscribe voluntarily through an email address and to provide background characteristics for study purposes. After 2 months, when the number of recommendations started to increase, patients and patients’ representatives requested us to add 2 sections to the existing structure of the wiki: 1 regarding the care delivered by the laboratory (eg, recommendations regarding semen analysis), and 1 regarding the care delivered by the remaining professionals who were not represented in a separate section (eg, recommendations regarding the medical psychologist). Hence, we added 2 sections to the wiki: laboratory and remaining. Next, we recategorized recommendations from the general section regarding care delivered by the laboratory or care delivered by professionals other than the general practitioner, gynecologist or urologist. After this restructuring of the wiki, the general section contained only recommendations on fertility care in general, thus not referring to the care delivered by 1 of the professionals involved.

##### Modifying Recommendations

After 7 months, we modified the recommendations in several steps. First, we removed duplicate recommendations. Then, if necessary, we moved recommendations into the appropriate sections (EB, JK). Since all recommendations in the remaining section turned out to be more suited to other sections, we eliminated this section. Next, 2 researchers (EB, WN) and the chief executive of Freya (JK) independently assessed the implementability of all recommendations using the Guideline Implementability Appraisal (GLIA) instrument [30]. Discrepancies were discussed and resolved through consensus. Based on the results of this assessment, the recommendations were independently textually refined or modified by a researcher (EB) and the chief executive of Freya (JK). Finally, after consensus was reached on the final formulation, we reentered the recommendations into the wiki.

##### Prioritizing Recommendations

All patients visiting the wiki website were invited to prioritize their top 5 (modified) recommendations in each section (for the laboratory section, we asked them to identify their top 3 due to the small number of recommendations). This prioritization was privately conducted by assigning 5, 4, 3, 2, and 1 points for the most important recommendations for determining high-quality fertility care for each of the 5 sections and independently from the subsections.

##### Assessing Eligibility of the Selected Recommendations

Initially, the CPG development group had intended to integrate this final top selection of patients’ recommendations directly into the CPG. However, before integrating these recommendations, the entire CPG development group (n = 11) assessed the eligibility of the recommendations for inclusion in terms of the scope of the guideline.

### Evaluation of the Wiki

To evaluate the feasibility of the wiki, we performed a multimethod evaluation study including three components [31]. First, to assess the ability to involve large and diverse patient populations compared with other methods such as interviews, we evaluated wiki use and users’ characteristics. Second, we evaluated wiki content quality (ie, recommendations) and, third, wiki system quality (eg, ease of use, layout), identifying factors that could potentially influence adoption of the wiki (barriers and facilitators) as well as potential factors for improvement.

#### Evaluation of Wiki Use and Users’ Characteristics

Data on actual use of the wiki (eg, number of unique visitors, page views) were generated through log files on the website of the patient association (Freya). Unique visitors were determined by IP address logged and stored on the website.

#### Evaluation of Wiki Content Quality

To evaluate the content quality of the wiki, we assessed various aspects of the final set of unique recommendations, particularly the number of recommendations, their content, and their eligibility for integration into the CPG for high-quality fertility care.

#### Evaluation of Wiki System Quality

To evaluate the quality of the wiki system and to identify facilitators, barriers, and potential areas of improvement, we conducted an online questionnaire. To gain insight into the thoughts underlying the resulting factors that formed potential facilitators of or barriers to adoption of the wiki and aspects of improvement, we conducted in-depth interviews with wiki users who completed the evaluation questionnaire.

#### Online Evaluation Questionnaire

During the prioritization phase, patients visiting the wiki website were invited to complete an online evaluation questionnaire. This questionnaire included items regarding users’ background characteristics (eg, age, type of infertility), use of the wiki (eg, number of visits), and factors that could potentially influence adoption of the wiki (quality of the wiki website, satisfaction, and net benefits) based on the relevant evaluation factors derived from the Human, Organization, and Technology-fit framework [32]. Questions on the potential influencing factors were grouped into 5 sections: ease of use of the wiki website, layout of the wiki website, value of the wiki methodology as a participatory tool for CPG development, content of the wiki website, and experienced privacy on the wiki website. Patients were asked to rate 22 accompanying positively formulated statements on these factors on a 5-point Likert scale from 1 (strongly disagree) to 5 (strongly agree) (Multimedia Appendix 1). After each section, patients were invited to comment. Next, patients were asked to describe their three advantages and disadvantages of the wiki website and potential areas of improvement. Finally, patients were asked for their willingness to participate again in a similar project and for their intention to recommend this wiki.

#### In-Depth Interviews With Wiki Users

We first summarized the identified influencing factors on adoption of the wiki website and suggested potential areas of improvement. Next, we translated these into a topic list to guide the in-depth interviews. To get both confirmation of and saturation in the thoughts underlying the facilitators of and barriers to adoption and potential areas of improvement of the wiki, 1 researcher (EB) conducted semistructured in-depth interviews with wiki users by telephone. Participants in the questionnaire survey who left their email address were randomly recruited by email. The first part of the interview consisted of open-ended questions, related to thoughts underlying the identified influencing factors on adoption and potentials for improvement of the wiki. Next, patients were asked for additional influencing factors and suggestions for improving the wiki. Recruitment continued until saturation of data was achieved. Regarding the starting and stopping criteria according to Francis and colleagues [33], we started with 2 interviews and aimed to repeat cycles of 2 interviews until we obtained no new data. If data saturation was achieved, an additional interview was conducted to attain data saturation.

### Data Analysis

We used SPSS 16.0 for Windows, Data Entry 4.0 (IBM Corporation, Somers, NY, USA) to perform descriptive statistical tests on the background characteristics of the wiki participants and to analyze patients’ top rankings of the recommendations. The final top selection of recommendations in each section was determined by identifying those with the highest sumscores derived. For analyzing the results of the online evaluation questionnaire, we grouped the responses on the 5-point Likert scale into the categories agree (scores 1and 2), neutral (score 3), and disagree (scores 4 and 5). Items were a priori identified as facilitators of adoption if >50% chose agree (scores 1 and 2) and as barriers to adoption of the wiki website if >50% of the evaluators chose disagree (scores 4 and 5). We used the reported top three advantages and disadvantages and the potential areas of improvement of the wiki to determine the frequency of occurrence of each aspect. We conducted an initial content analysis of all free-text responses to the questionnaire, to determine additional points to be improved (EB, WN).

#### Qualitative Analysis of the Interviews

All interviews were audiotaped and transcribed verbatim. Data were analyzed iteratively and thematically across accounts (EB, JK) [34], according to the relevant factors of the evaluation framework, as used in the questionnaire to identify barriers to and facilitators of adoption and potential areas of improvement of the wiki [32]. Another researcher (WN) independently checked the coding framework and analysis.

## Results

### Wiki Tool Development

#### In-Depth Interviews

**Figure 1:**
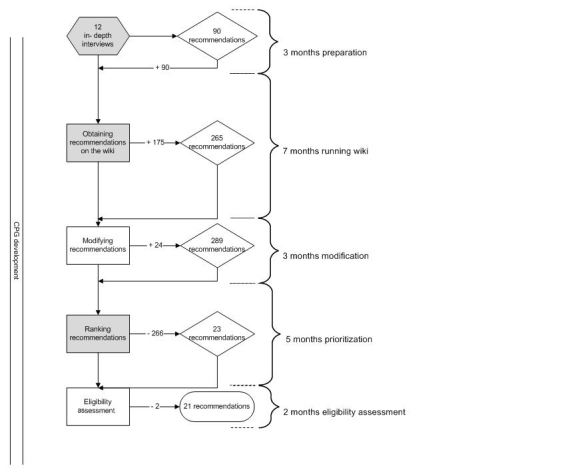
The process of obtaining recommendations for clinical practice guideline (CPG) development.

From the transcripts of 12 in-depth interviews with infertile patients, we translated the perceived bottlenecks into a set of 90 draft patient recommendations and entered them into the wiki (Figure 1).

For example, patients perceived a bottleneck in that appointments were possible only during working hours instead of also during the evening, which resulted in difficulties with work. The resulting draft recommendation was formulated as “I want the hospital to provide possibilities to make appointments during evening hours.” Other examples of the bottlenecks mentioned were the variation between hospitals’ laboratories in performing a semen analysis, unavailability of separate waiting rooms for pregnancy and infertility consultations, and gynecologists’ lack of empathy.

#### Structure of the Wiki

**Figure 2:**
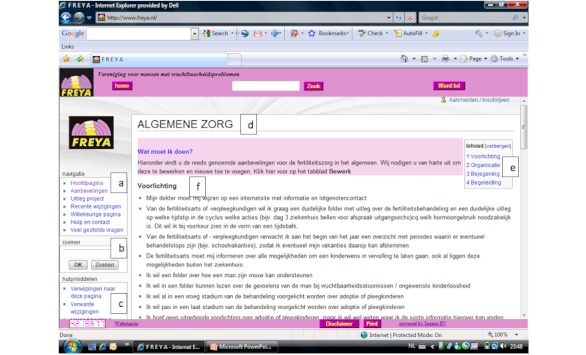
Screenshot of recommendations on FreyaWIKI.

**Figure 3:**
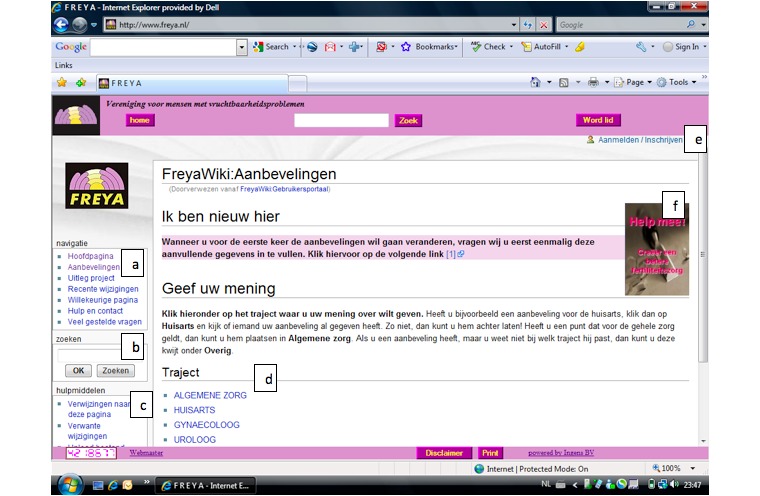
Screenshot of the FreyaWIKI homepage.

FreyaWIKI was structured through the division of recommendations into 6 sections. Each of these sections was subdivided into 8 subsections (Figures 2 and 3, see Multimedia Appendices 2 and 3 for translations).

### Patient Participation in CPG Development

#### Wiki Use and Users’ Characteristics

**Figure 4:**
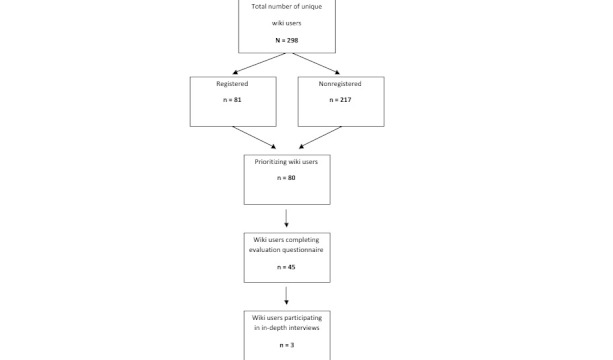
Flow of wiki participants through the study.

During 7 months of access, 36,473 wiki pages were viewed. We identified 298 unique users, including 81 registered users who provided background characteristics (Figure 4). The majority of them were female 78/81 (96%), highly educated 54/81(67%), and middle aged (mean 33 years). Median duration of infertility was 30 months (range 0–71 months). More than half 43/81(53%) underwent medically assisted reproduction techniques during the period of their visit. Another 14% (n = 11) stayed childless despite treatment.

#### Wiki Content Quality

Overall, we collected 265 recommendations and modified them into 289 unique recommendations (Figure 1). After patients (n = 80) prioritized the recommendations by ranking the top 5 or top 3 in each section (we had eliminated the remaining section) according to their importance to high-quality fertility care, we selected 23 recommendations (4 sets of top 5 and 1 set of top 3) for eligibility assessment by the CPG development group (Table 1). We excluded 2 insurance-related recommendations, since they did not meet the scope of the CPG. The CPG development group accepted all of the remaining 21 recommendations, which were directly integrated into the CPG. More than half (n = 11) of the final set of recommendations concerned the organization of care. Similar to the quality assessment scale used in evidence-based recommendations (levels of evidence A-D) [35], a level of P (patients) was provided for the patients’ recommendations and formulated as “ Patients would like to….” Participants were informed by email, on the wiki website, and through the websites of Freya and the professional associations of the final CPG that included the untouched eligible recommendations of the patients.

#### Evaluation of Wiki System Quality

##### Online Evaluation Questionnaire

Of the 80 patients who participated in the prioritization, 45 completed the questionnaire. Of these, 53% (n = 24) visited FreyaWIKI for the first time while completing the questionnaire, and 93% (n = 42) had never worked with a wiki, other than this one, before. Other background characteristics of the respondents are presented in Table 2. Facilitators of adoption of the wiki, defined as >50% of respondents agreeing (scores 1 and 2) to the relevant statements, were not identified. Barriers, defined as >50% disagreeing (scores 4 and 5) to the relevant statements, were identified in 3 of the 5 sections: ease of use, content of the website, and value of the wiki methodology (Table 3). In decreasing order of the proportion of evaluators who disagreed with the relevant statement, the main identified barriers concerned the findability (82%) and accessibility (78%) of the website, and the suitability of this wiki for obtaining recommendations for CPG development (71%).

Reported advantages of the wiki were the privacy they experienced on the website, the structure of the website linking recommendations to sections on care delivered by fertility professionals, ease of navigation through the website, and the additional value of the wiki website as a source of information and as an opportunity to provide feedback to the care services.

Reported disadvantages of the wiki concerned the content of the wiki website, in terms of the unstructured recommendations not being formulated in a similar way, too much content being visible on one screen, and the nonattractive layout of the wiki website.

The main potential areas of improvement were providing information on treatment options and causal factors of infertility on the wiki website, broadening the marketing of the wiki by placing advertisements in commercial magazines, and communicating information on related activities (Table 4). Overall, 98% of the patients said they would recommend the website and 84% would participate again in a similar project.

##### In-Depth Interviews

Overall, 11 of the 30 patients who gave their email address in the evaluation questionnaire agreed to participate in the interviews. We conducted 3 interviews. All 3 interviews confirmed barriers to adoption as well as suggestions to improve the wiki, and saturation of the related underlying thoughts was reached (Table 4). All patients reported problems with formulating a recommendation and expressed their wish to add a personal touch to the recommendation (eg, to explain why something should be done). The introduction of a motivation page, where patients could describe why they formulated a recommendation, might meet this request. Patients also embraced the missing community feeling as mentioned in the evaluation questionnaire. Introducing a monthly newsletter and automatically sending an email to the person who made the recommendation were suggested. All 3 interviewees regarded the website as a valuable source of information, rather than as a tool for modifying recommendations for CPG development. They mentioned that the content of the wiki had been helpful to them in searching for information on experiences regarding infertility treatment and in searching for recognition of their own experiences. Challenges faced by users in understanding the purpose of the website would be addressed by clearer instructions.

**Table 1:** Final set of the patients’ top-5 and top-3 recommendations (n = 23) for the 5 sections, ranked by importance to the quality of fertility care as formulated on the wiki website.

Section, rank, and recommendation			Subsection (aspect of care)^a^
**General care**			
	1	I want insurance companies to reimburse six attempts at in vitro fertilization^b^	General
	2	I want insurance companies to start counting in vitro fertilization attempts only after oocyte retrieval or even after embryo transfer has been performed^b^	General
	3	I want my doctor to practice empathy, instead of only working on the technical or financial part	Staff attitudes
	4	I want the hospital to have separate waiting rooms for pregnant women and couples being treated for infertility	Organization
	5	I want to be able to arrange appointments during the daytime as well as in the evenings	Organization
**General practice care**			
	1	I want my gynecologist and GP^c ^to have good communication, so my GP knows what is going on with us	Referral
	2	I want my GP to make a referral immediately after we have been trying to conceive for a year	Referral
	3	I want to have my first medical consultation with my gynecologist within 1 month after referral.	Organization
	4	I want my GP to be informed of possible causes of infertility, in both women and men	General
	5	I want my GP to pay attention to nonmedical issues, such as stress, anxiety, relational problems, and sexuality	Communication
**Gynecologic care**			
	1	I want also to be able to receive treatments on weekends (Saturdays and Sundays)	Organization
	2	I want all members of the fertility treatment team to apply one policy regarding my infertility treatment	Organization
	3	I want my gynecologist to inform me of all possible fertility treatment options, even if these are outside the hospital	Information provision
	4	I want my gynecologist to inform me about the different phases of treatment and their expected time span	Information provision
	5	I want assisted hatching to be possible or available in the Netherlands	Therapy
**Urologic care**			
	1	I want my urologist and gynecologist to have good communication	Organization
	2	I want to be informed of the investigations that are to be performed by the urologist	Examination
	3	I want to have a permanent urologist who is specialized in infertility	Organization
	4	I want to have a consultation with a urologist within 1 month after referral	Organization
	5	I want my urologist to involve my partner in the conversation	Communication
**Laboratory**			
	1	I want to be informed as soon as possible when our embryos do not divide correctly	Organization
	2	I want Dutch laboratories to share protocols and learn from each other’s experiences	Organization
	3	I want to be informed of the causes of nonviability of our frozen embryos, if appropriate	Organization

^a ^Subsections were derived from the website’s structure and defined by the user.

^b ^Recommendation was excluded, since it fell out of the scope of the clinical practice guideline.

^c ^General practitioner.

**Table 2:** Background characteristics of respondents (n = 45) to the evaluation questionnaire.

Characteristic		Data
**Gender, n (%)**		
	Male	0 (0%)
	Female	45 (100%)
Age (years), mean (SD)		35 (5.24)
**Type of infertility, n (%)**		
	Primary	15 (33%)
	Secondary	30 (67%)
Duration of infertility (months), median (range)		36 (0–71)
**Current phase in fertility care, n (%)**		
	Gynecologic	19 (42%)
	No pregnancy after fertility treatment	8 (18%)
	Pregnant achieved by fertility treatment	4 (9%)
	Unknown	14 (31%)
**Level of education, n (%)**		
	Low	0 (0%)
	Intermediate	14 (31%)
	High	31 (69%)
Membership in Freya, n (%)		24 (53%)

**Table 3:** Patients’ barriers to adoption of the wiki (n = 45).

Factor influencing adoption of the wiki		Proportion disagreeing with the factor^a^	
		n	%	
**Ease of use of the website**			
	Findability of the website	37	82%
	Accessibility of the website	35	78%
	Clarity of log-in location on the website	27	60%
	Clarity on the goal of the website	28	62%
	Clarity on instructions for using the website	24	53%
	Efficiency of the website (ie, speed at which the website enabled users to accurately and successfully add and modify recommendations)	24	53%
**Content of the website**			
	Comprehensiveness of the clarifying text on the website	30	66%
	Satisfaction with the content of the formulated recommendations	25	56%
	Usefulness of clustering recommendations into sections in searching for existing recommendations	23	51%
	Similarity between formulated recommendations and participants’ actual opinions on fertility care	23	51%
**Value of using the wiki**			
	Suitability of the wiki for obtaining recommendations for clinical practice guideline development	32	71%
	Ease of using the wiki	24	53%
	Accessibility of the wiki	27	60%

^a ^Number (%) of participants who rated the positively formulated statements on the evaluation factors as disagree (scores 4 or 5).

**Table 4:** Participants’ (n = 45^a^) suggestions for improving the wiki website.

Aspect of improvement		Respondents suggesting the aspect		Sample quotes (translated from Dutch) from in-depth interviews (I) and online questionnaires (Q)
		n	%	
**Usability of the website**				
	Findability of the website	10	22%	Q:*Hard to find* Q: *I think it is awkward that the website is only findable through the Freya website* I: *I wouldn’t know how to find the website, unless through the Freya website*
	Accessibility of the website	2	4%	I: *I was unable to find the log-in location or request a new password*
**Content of the website**				
	Comprehensiveness of clarifying text	1	2%	Q: *Unclear*
	Clearness of description of the goal of the wiki	4	8%	I: *The description is a bit unclear; therefore, I previously thought to check it more precisely, but I still haven’t done this* I: *I had not concluded that the recommendations were directly integrated in a professional guideline*
	Clearness of instructions for use	1	2%	
	Satisfaction with formulated recommendations	8	16%	I:...*but there are recommendations I am not satisfied with, I would suggest that participants can prioritize recommendations that they are satisfied with in an earlier stage, then you only have to list the most important recommendations in one screen*
	Similarity between actual preferences and recommendations	4	8%	Q: *I would like to see why a specific recommendation was formulated, separately from the recommendation* I: *There are too many recommendations on the website, but there are recommendations I am not satisfied with. I would suggest that participants can prioritize recommendations that they are satisfied with*
	Clarity of the structure in which recommendations are placed on the website	30	66%	I: *Structure is good but the provided sections are incomplete, for example the care provided by a psychologist or other forms of mental counseling. Psychosocial concerns are always underestimated in fertility care* Q: *The used structure is good, but for searching an existing recommendation it would be valuable to add a search function to the website*
	Relationship between length and number of recommendations and their presentation on one screen	32	71%	Q: *There are too many recommendations on the website* I: *Recommendations are too long, sometimes it’s more like a story, which is very interesting, but I wonder if the doctors are taking this as serious input to a guideline* Q: *The prioritization is hard due to the large number of recommendations*
	Education provision on the website	19	42%	Q: *It might be valuable if the website provides usable links to high-quality websites* Q: *Information on treatment options might enrich the website* Q: *I would like to find information on causal factors of infertility* Q: *Practical information about compensations for treatment per insurance company, regional psychological services, plural miscarriages, infertility, and referral*
**Characteristics of the wiki**				
	Usability of wiki methodology	6	13%	Q: *The website is not user friendly...the number of visible recommendations makes it unclear* *Q: Recommendations given contain too many words* I: *I really don’t have a clue about what constitutes a high-quality recommendation* I: *It would be valuable to apply an automatic program, through which patients are able to formulate recommendations*
**Accessibility of wiki methodology**				
	Efficiency of wiki methodology	5	11%	Q: *Prioritizing is hard and not efficient in this stage, the list of recommendations is too long* I: *The efficiency might be improved if you ask patients immediately after formulating a recommendation to prioritize the most important recommendations*
**Layout of the website**				
	Impression of the layout	33	73%	Q: *Nonattractive/not a modern/not a fashionable website* Q: *The layout is not from today* Q: *Looks unprofessional*
**Communication with wiki users**				
	Marketing	6	13%	Q: *This good initiative requires a better marketing approach to reach more participants*
	Community feeling of the wiki	3	6%	I: *More communication on related activities and results will increase the number of patients that will come back* Q: *Effect of the recommendations on the guideline is unclear*

^a ^45 participants completed the online evaluation questionnaire, of whom 3 participated in the in-depth interviews.

## Discussion

### Principal Results

In this study, we showed that the wiki is a feasible tool to ensure active patient participation in the development of a Dutch multidisciplinary CPG on infertility. The high numbers of page views (36,473), unique visitors (298), and recommendations formulated (289) implies patients’ willingness and ability to contribute to CPGs through a wiki-based method. We also showed that such a wiki is a useful information source for patients.

Second, we gained a final set of 21 selected recommendations, which were assessed as being eligible to be integrated directly and transparently into the CPG. Third, patients had positive views on the experienced privacy, ease of navigation, divisional structure of the wiki, and its potential befits. A total of 98% of the patients would recommend the website and 84% would participate again in a similar project. This study also provided some important suggestions to improve this participatory tool for patients in the development of CPGs, concerning ease of use, content and layout of the website, and characteristics of the wiki tool.

### Comparison With Existing Techniques

Several studies on specialized medical wikis (eg, wikis that fall outside the scope of a general encyclopedia) have been published, but most particularly focus on education of medical students [36] or collaboration between health care professionals [37,38], rather than on patients, and did not include a process evaluation. Only Gupta and colleagues [39] and Archambault [40] involved a group of preselected patients as well as professionals in the development of an asthma action plan through a wiki. However, results are premature, since this study was conducted over a very short time period (weeks), and a wiki needs more time to build content (approximately 7-8 months) [41]. Furthermore, Gupta and colleagues’ and Archambault’s evaluation of the wiki tool was not focused on patients’ experiences and was less extensive than our multifaceted approach to gaining insight into patients’ barriers to adoption of our wiki. In this study, we involved a large number of patients (298), which cannot be realized using traditional methods, such as focus groups, in which participation is generally restricted to a maximum of 8 participants [42]. We even assessed the final selection of top recommendations for their eligibility for direct integration into the CPG. Thus, the patients’ contribution to the CPG was clearly illustrated by integrating their recommendations in their entirety, indicated by the new P level (Patients). We also addressed other practical limitations of the methods used to enhance patient participation in CPG development, such as organizational (eg, recruitment of participants), financial (eg, travel costs), and sociopolitical (eg, professional resistance to including patients in CPG development group) constraints.

Professionals and patients’ representatives could also use the wiki and had the opportunity to informed themselves about patients’ views and to bring up content for discussion in the CPG development group. According to the results of the evaluation questionnaire and the interviews, this content was also helpful to patients as an information source, which may also explain the relatively large number of page views. Although providing information was not the initial goal of this wiki, its relevance is in agreement with published literature on conventional wikis [19] and with European patients’ perception of the importance of the Internet as a source of information [43]. Hence, this unintended consequence concurrently yields challenges for improvement and might be aided by providing clearer instructions for use and description of the goal of the wiki, but also addresses important implications for future studies in this field. Next to the informational value of formulated recommendations for high-quality care, attention should be paid to useful links to relevant websites that may potentially attract more patients to the wiki website and increase the chances for adoption of an improved version of the wiki.

Although drawbacks to active patient participation methods were reduced, this study drew attention to some other potential implications derived from patients’ suggestions that might improve the use of a future medical dedicated wiki exclusively for actively involving patients in CPG development. First, structuring recommendations and limiting the number and length of recommendations to presentation on one screen may improve usability [44]. Second, using a fixed format in the formulation of recommendations, based on relevant items of the GLIA instrument, may not only improve usability and accessibility of the wiki [30,44], but may also improve the efficiency of the wiki and the usefulness of recommendations in being integrated directly into the CPG. Introducing a motivation page might give patients the opportunity to add a personal touch to the recommendation. Third, a prioritization system, continuously refining the similarity between patients’ perspectives and the top5 recommendations (eg, by rating recommendations after every contribution), could improve the tools’ efficiency by avoiding separate prioritization of recommendations and could improve patients’ satisfaction with the highest-rated recommendations. This modality would also allow more flexible use by CPG developers at the time of their choosing. In addition to the suggested modalities, some known refinements in overall usability (eg, findability, prominent log-in location), content (comprehensiveness of text), and layout of the website might improve use of the wiki and would be reduced by repeated cycles of design, evaluation, and redesign [45,46]. Furthermore, a user-centered design, in which patients codevelop such new modalities, may improve future implementability and provide chances for local adaptation of a redesigned wiki website [47,48]. Both the feasibility of a wiki as a participatory tool for patients in the development of CPGs and the recommendations for future wiki-based initiatives illustrate the value of eHealth. With this in mind, numerous participatory applications based on wikis are conceivable and may be valuable in various fields of research. In the field of guideline development, guideline-derived initiatives actively involving patients in the development of patient information leaflets or treatment action plans, in addition to fully online-based CPGs, may also benefit from our results. Finally, our results add to the knowledge base about wikis in health care [49].

### Limitations

This wiki has been tested in the field of infertility care, representing a relatively young target group [50]. More than 98% of this group use the Internet [21]. This participant characteristic is associated with more frequent health-related Internet use [51-53]. Therefore, the participants in our study were an ideal subgroup for testing and evaluating a wiki-based method, which argues against the generalizability of our findings to other patient groups. Nevertheless, health-related Internet use in Europe is increasing over time [54]. Hence, it seems to be a question of time until older people or their caregivers, or both, will be using such tools [55]. Furthermore, this feasibility study provided an important exploratory evaluation component, which resulted in valuable information for future studies in this field but also had certain limitations. First, based on the results of a recent systematic review from Gagnon and colleagues [56], we acknowledge that the items used in our evaluation questionnaire might be incomplete. However, the results of our study add to those from the limited number of previously published studies on patients’ facilitators of and barriers to adoption of eHealth applications [57,58]. Second, the heuristics used were not based on a validated questionnaire and were too limited for drawing conclusions on the usability that patients perceived. Therefore, a next step in future development of a wiki-based participatory tool for patients in CPG development should be to include a broader evaluation of the potentially influencing factors on adoption, including more organizational factors and a heuristic evaluation.

Third, the participation rate in the evaluation of the wiki might have subjected our study to a participation bias of potentially the most motivated wiki users. However, this is a known limitation in the active use of wikis in general: the most motivated users provide most of the content [19]. Finally, this feasibility study did not assess the representativeness of either the participants or the final set of recommendations in the wiki.

### Conclusions

The wiki is a promising and feasible tool to actively involve patients in CPG development. To improve the tool’s ease of use and practical aspects to enhance direct integration of recommendations into the CPG, a more specialized and refined wiki should be developed. This should include new modalities, such as automatically shortening the number and length of recommendations, using a fixed format for formulation of recommendations, using a continuous prioritization system for selection of the most important recommendations, and including a separate motivation page. Furthermore, in the development, attention should be paid to the informational character of such a wiki. To improve future implementability, a modified tool should preferably be codeveloped and evaluated by patients in a user-centered design study. Furthermore, representativeness of patients and recommendations should be integrated into this next phase.

## Multimedia Appendix 1

Constructs of the online evaluation questionnaire.

## Multimedia Appendix 2

Screenshot of recommendations on FreyaWIKI.

## Multimedia Appendix 3

Screenshot of the FreyaWIKI homepage.

## Abbreviations

AGREE: Appraisal of Guidelines Research and Evaluation
CPG: clinical practice guideline
GLIA: Guideline Implementability Appraisal
