# Supplementary material for: Feasibility of a Wiki as a Participatory Tool for Patients in Clinical Guideline Development
Source: J Med Internet Res. 2012 Oct 26;14(5):e138. doi: 10.2196/jmir.2080 (PMC3510744; doi:10.2196/jmir.2080)
Supplement: Supplementary file 1 [file jmir_v14i5e138_app1.pdf]

Statements (n=22) rated on a 5-point Likert scale (1= strongly agree, 5= strongly disagree)
**Ease of Use of the website**
□ I am able to find the website easily<br>□ The website has a good availability<br>□ The website has a clear place for login<br>□ The goal of the website is clear to me<br>□ The instructions for use of the website are clear to me<br>□ The instructions for modifying previously formulated recommendations and adding new recommendations are clear to me<br>□ The use of the website is efficient (speed at which the website enables a user to accurately and successfully add and modify recommendations)<br>□ Navigating through the website is easy<br>□ The links on the website are functional<br>□ It is clear to me where to ask questions and/or report problems with the website
**Layout of the website**
□ The used layout makes the website accessible<br>□ The used layout encourages the use of the website<br>□ The layout of the website is conveniently arranged
**Content of the website**
□ The content on the website is well organized<br>□ The clarifying text on the website is easily understandable<br>□ I am satisfied with the content of the recommendations formulated on the website<br>□ The clustering of recommendations into different sections on the website is useful to search recommendations (general care, gynecologic care etc.)<br>□ The recommendations formulated on the website are in agreement with my actual opinion on fertility care in the Netherlands
**Value of the used wiki**
□ The used wiki is a suitable way of gaining recommendations for a national guideline<br>□ The used wiki is easy in use<br>□ The used wiki is easy accessible
**Privacy on the website**
□ I am not afraid for abuse of my personal data on the website
**Additional open questions**
o Can you provide the three advantages of the website?<br>o Can you provide the three disadvantages of the website?<br>o Can you provide potentials for improvement of the wiki-website?<br>o Would you recommend wikifreya to others?<br>o Would you re-participate in a similar project in the future?<br>o Do you have any additions to his questionnaire?

□ = statement

o = open question
