# Supplementary material for: Feasibility of a Wiki as a Participatory Tool for Patients in Clinical Guideline Development
Source: J Med Internet Res. 2012 Oct 26;14(5):e138. doi: 10.2196/jmir.2080 (PMC3510744; doi:10.2196/jmir.2080)
Supplement: Supplementary file 2 [file jmir_v14i5e138_app2.pdf]

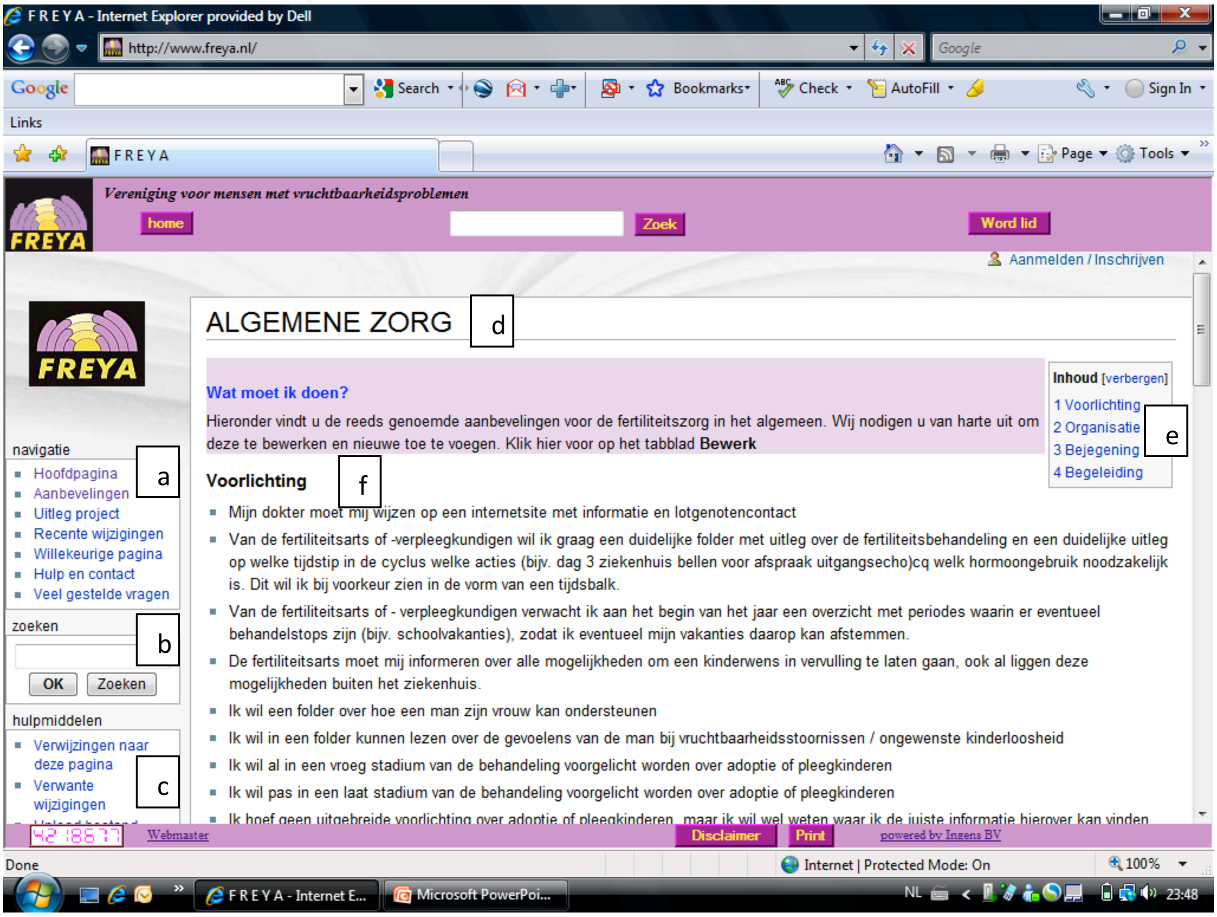

a= Navigation: homepage, recommendations, project description, recent modifications, random page, help and contact, frequently asked questions.

b= Search

c= Help

d= Section General care

e= Subsections regarding information, organization, staffs' competence and communication.

F= Recommendations on section general care and the subsection information provision.

Translation:

GENERAL CARE

What should I do?

Below you find the recommendations for fertility care in general. We kindly invite you to modify these or add new recommendations. Click *here* to edit.

Information provision

* My doctor needs to point out the internet sites providing information and contact with co-patients.

* etc.
