# Supplementary material for: Feasibility of a Wiki as a Participatory Tool for Patients in Clinical Guideline Development
Source: J Med Internet Res. 2012 Oct 26;14(5):e138. doi: 10.2196/jmir.2080 (PMC3510744; doi:10.2196/jmir.2080)
Supplement: Supplementary file 3 [file jmir_v14i5e138_app3.pdf]

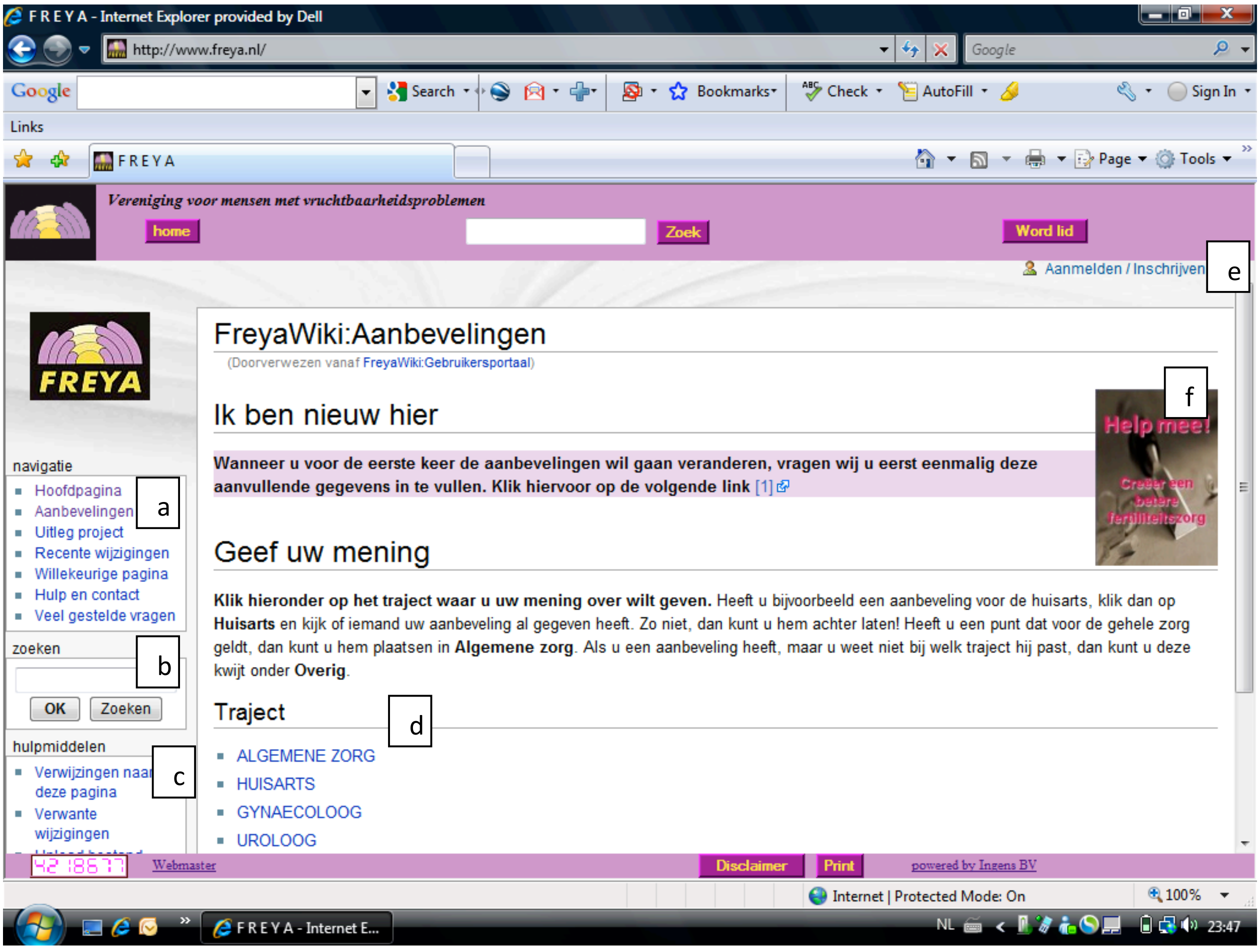

a= Navigation: homepage, recommendations, project description, recent modifications, random page, help and contact, frequently asked questions.

b= search

c= help

d= listed sections

e= button to register

f= image of the marketing poster used in waiting rooms of all Dutch gynecologists and Urologists

Translation:

FreyaWiki: Recommendations

I am new here

If you are planning to edit the recommendations, please register and fill in your background characteristics. Click on the following link.

Share your ideas

Click below on the section, of which you would like to share your ideas. For example, you have a recommendation for the General Practitioner, then click on **General practitioner** and look if your recommendation has been previously formulated by someone else. If not, you may add your recommendation. If you have a recommendation for the entire fertility care, then you can leave it in the **General Care** section. If you have a recommendation, of which you don't know to which section this applies, you may add this to the section **Remaining**.

Sections

* General care

* General Practitioner

* Gynecologist

* Urologist
